# Editorial: Treatment resistance in psychotic disorders

**DOI:** 10.3389/fpsyt.2023.1181914

**Published:** 2023-03-21

**Authors:** Santanu Nath, Eugenia Kravariti, Rajeev Ranjan, Venkata Lakshmi Narasimha

**Affiliations:** ^1^Department of Psychiatry, All India Institute of Medical Sciences, Deoghar, Jharkhand, India; ^2^Institute of Psychiatry, Psychology and Neuroscience, King's College London, London, United Kingdom; ^3^Department of Psychiatry, All India Institute of Medical Sciences, Patna, Bihar, India

**Keywords:** psychosis, schizophrenia, treatment resistance, clozapine, ECT

Psychotic disorders are severe mental disorders that have a devastating impact on the socio-occupational functioning of the sufferer. Psychosis, the major symptom construct of these disorders, can however arise from several medical, neurological, developmental, and psychiatric conditions (1). The core symptoms of psychosis include hallucinations, delusions, disorganized thinking, speech, and motor behavior (including catatonia), and negative symptoms, which can significantly hamper reality testing and impinge strongly on the daily functional aspects of normal life (2).

Psychotic disorder is an umbrella term that has within its rubric disorders like Schizophrenia, brief psychotic disorder, schizophreniform disorder, delusional disorder, schizoaffective disorder, and also psychosis in the context of substance use disorders, medication use, and medical causes (1, 2). The majority of psychotic disorders require long-term treatment. Pharmacological therapy with antipsychotics takes the center stage in their management, while psychotherapies also supplement them to constitute what is described as the holistic care in the management plan of psychotic disorders (3). Antipsychotics have evolved ever since chlorpromazine and clozapine were discovered in the 1950s. Over the years, various types and chemical classes of antipsychotics have been discovered and they have been used for the treatment of schizophrenia, in particular, and psychosis in general with varying response rates. While the dopamine hypothesis of psychosis was the leading theory to guide the development of antipsychotics, newer lines of evidence have also incorporated the role of serotonin, GABA, glutamate, and even nicotinic cholinergic neurotransmission in the pathogenesis of psychotic disorders, thus paving the way to newer antipsychotics with fewer adverse reactions, albeit with varying degrees of success (4). To this day, managing psychosis is a challenge to psychiatrists worldwide, and the psychopharmacological armamentarium to treat psychotic disorders is of little success. Around 30% of the sufferers of psychosis continue to have significant morbidity and disability after standard antipsychotic treatment, i.e., they develop treatment resistance (5). Treatment resistance in psychosis is defined as inadequate therapeutic response to at least two adequate trials of antipsychotics. This leads to significant impairment in the individual sufferer and inflates healthcare utilization and costs of treatment (6). Till date, clozapine has been the only antipsychotic approved for treatment resistant schizophrenia (a prototype of psychosis), but it is associated with some common and serious adverse effects (7). When clozapine fails, the management becomes more difficult, and this is associated with a poorer outcome.

Treatment resistance in psychotic disorders thus poses significant challenges both to sufferers and to mental health professionals. Since the gamut of psychotic disorders is large as far as individual diagnostic groups in nosology are concerned, no particular management plan of treatment resistance for one group of psychosis is a good fit for others. There are putative differences in our neurobiological understandings of these different psychotic disorders and also their treatments. The current Research Topic attempted to address treatment resistance in psychosis (TRP) from a holistic perspective. Researchers were invited to submit papers addressing different aspects of TRP, and the current topic collection mirrors their submissions. Total six submissions were received of which two are original research, one brief research report, two mini reviews and one perspective article.

Of the two original research articles, the first by Samuel et al. was undertaken in a psychiatric institution in Ethiopia, wherein the authors aimed to assess the association between social support and psychotic relapse in patients with schizophrenia. They recruited 396 patients with schizophrenia and involving a case-control model, they found social support to be a significant contributor of schizophrenia relapse with poorer support being associated with higher odds of relapse (odds ratio: 3.102). The authors emphasized that both family members and mental hospitals should increase support to patients with schizophrenia that has a bearing on the prognosis. The second by Lappin et al. was conducted in Australia, wherein the authors assessed how much of the recommended treatment options (clozapine and/or ECT) for treatment resistant psychosis (TRP) is actually practiced in a real-world situation. The authors reported that though around 89% of those who are fulfilling criteria for TRP had received clozapine at some point, only 39% of them are currently being prescribed the drug. The utilization of ECT (25%) was however poorer than clozapine. They also noted that a full trial of clozapine was seldom attempted (median trial duration: 4 months) and clozapine plasma levels were also rarely measured. They concluded with a note that there need to be strategies to optimize the utilization of clozapine and ECT for those with TRP for adequate therapeutic effectiveness and overall wellbeing.

Zheng et al. in their brief research report that attempted to assess the prescribing practices of psychiatrists of Hong Kong and Singapore in treatment resistant schizophrenia (TRS) and clozapine resistant schizophrenia (CRS), found that although more than 88% of the respondents were aware of the management guidelines for the condition being studied, nearly half of them delayed clozapine in TRS when adequately indicated. As an alternative, half of them would use high dose of non-clozapine antipsychotics, depot antipsychotics, or antipsychotic combinations, and also adjunct mood stabilizers. These alarming figures again reiterate the finding by Lappin et al.. The authors concluded that factors contributing to the well-documented delay in clozapine prescription need to be understood, and further research is warranted to understand treatment options apart from clozapine.

Of the two mini reviews published, the first by Weston-Green discusses antipsychotic drug discovery and development over the years, and how and why even after 70 years of continuous advancements, treatment resistance is still a problem. She reiterated the need for development of novel molecules and focus on biotypes and biomarkers of schizophrenia to increase our knowledge of the pharmacological mechanisms of these novel compounds in better management of schizophrenia and TRS. The second review by Pattnaik et al. explores the diagnostic and management nuances of TRP in childhood onset psychosis (COP), and the use of clozapine in this population. The authors also provided a roadmap on the use of clozapine in COP, while also voicing a need for further research on TRP in children and consensus protocol for managing this disabling condition.

The single perspective article by Pandey and Kalita describes TRS from a holistic perspective starting from a historical evolution of the concept, the defining criteria for it, the proposed neurobiological underpinnings and the management options. The authors have stressed the need for precision medicine in understanding schizophrenia and TRS which may provide answers to the quest for discovery of novel treatment options for the condition.

Taken together, these articles have provided some light into the enigmatic concept of TRP and its management, while paving the pathway for future research on this area.

## Author contributions

The editorial was initially drafted by SN and EK, while VN and RR did the literature search. All authors have contributed in the overall editorial assignments. The manuscript before final submission has been seen, corrected, and finalized by all the authors.
